# The Root Clock as a Signal Integrator System: Ensuring Balance for Survival

**DOI:** 10.3389/fpls.2022.886700

**Published:** 2022-05-19

**Authors:** Estefano Bustillo-Avendaño, Laura Serrano-Ron, Miguel A. Moreno-Risueno

**Affiliations:** Centro de Biotecnología y Genómica de Plantas (Universidad Politécnica de Madrid – Instituto Nacional de Investigación y Tecnología Agraria y Alimentaria), Madrid, Spain

**Keywords:** biological clock, root branching, lateral root, oscillating gene expression, auxin signaling, retinal, water deprivation, heavy metals

## Abstract

The root system is essential for the survival of terrestrial plants, plant development, and adaptation to changing environments. The development of the root system relies on post-embryonic organogenesis and more specifically on the formation and growth of lateral roots (LR). The spacing of LR along the main root is underpinned by a precise prepatterning mechanism called the Root Clock. In Arabidopsis, the primary output of this mechanism involves the generation of periodic gene expression oscillations in a zone close to the root tip called the Oscillation Zone (OZ). Because of these oscillations, pre-branch sites (PBS) are established in the positions from which LR will emerge, although the oscillations can also possibly regulate the root wavy pattern and growth. Furthermore, we show that the Root Clock is present in LR. In this review, we describe the recent advances unraveling the inner machinery of Root Clock as well as the new tools to track the Root Clock activity. Moreover, we discuss the basis of how Arabidopsis can balance the creation of a repetitive pattern while integrating both endogenous and exogenous signals to adapt to changing environmental conditions. These signals can work as entrainment signals, but in occasions they also affect the periodicity and amplitude of the oscillatory dynamics in gene expression. Finally, we identify similarities with the Segmentation Clock of vertebrates and postulate the existence of a determination front delimiting the end of the oscillations in gene expression and initiating LR organogenesis through the activation of PBS in an ARF7 dependent-manner.

## Introduction

Living organisms produce periodic intermittent responses to generate biological structures and to adapt to cyclic environmental inputs. The ability of organisms to generate periodic responses relies on biological clocks or rhythms (Heltberg et al., 2021). Mathematically, the simplest way of generating periodic responses is through an oscillatory circuit containing a negative feedback loop and an in-built time delay in gene function such as the time required for transcription and/or translation among others (Rosier and de Greef, 2015). However, more complex oscillatory systems include both negative and positive feedback loops (Ray, 2008). Oscillators can be entrained to cyclic processes that can be either endogenous (coming from the organism itself) or exogenous (resulting from environmental cues). This entrainment process consists of the synchronization of one or more oscillatory rhythms with the cyclic endogenous or environmental signal (Roenneberg et al., 2005).

The universal example of a biological oscillator is the circadian clock, which is found in many kingdoms, including fungi, animals, and plants (Mcclung, 2006; Rosbash, 2009). Other oscillatory mechanisms only occur in certain types of organisms. In vertebrates, the process of somitogenesis, by which the paraxial mesoderm is segmented at regular intervals or somites to form the vertebral column, results from the activity of the Segmentation Clock (Linde-Medina and Smit, 2021). In Arabidopsis, the formation of lateral roots (LR) is driven by a prepatterning mechanism that results from the activity of the Root Clock (Moreno-Risueno et al., 2010). In this review, we compile the recent advances on the identification of the Root Clock activities and outputs, such as the generation of gene expression oscillations and the establishment of the branching locations, called pre-branch sites (PBS). Next, we describe the molecular core driving these activities, and how endogenous and exogenous signals can entrain the Root Clock or alter its periodicity and/or frequency. Based on available data, we discuss the role of the Root Clock as a signal integration system able to balance pattern conservation with plastic responses.

## Identification of New Root Clock Activities

**Figure 1:**
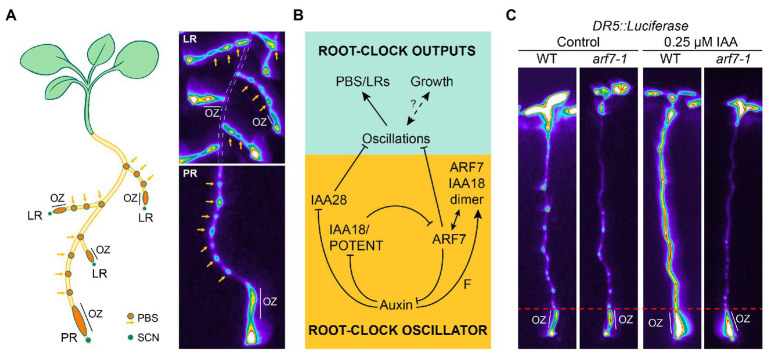
The activity of the Root Clock results from an inner oscillator. **(A)** Left: Schematic representation of the location of Oscillation Zone (OZ) and PreBranch Sites (PBS) in the root system. Right: Location of the OZ and PBS visualized by *DR5::Luciferase* expression in the primary (PR) and lateral roots (LR). Note that the LR (top right image) show OZ and form PBS the same as the PR (bottom right image). The PR is delimited by a dasshed white line in the top right image. SCN: Stem Cell Niche. **(B)** Model of the core genetic circuit driving the Root Clock as adapted from (Perianez-Rodriguez et al., 2021). ARF: Auxin Response Factor. IAA: Aux/IAA protein inhibitors. F: predicted factor promoting dimer formation. **(C)** *DR5::Luciferase* expression in 6 days post imbibition (dpi) plants treated with 0.25 μm auxin (indol-3-acetic acid, IAA) for 1.5 h. Note that activation of *DR5::Luciferase* occurs in the OZ of *arf7-1,* whereas it is impaired in the root region above as delimited by the dashed red line. This switch in behavior of *DR5::Luciferase* delimits the end of the Root Clock oscillations and marks the initiation of LR organogenesis through PBS activation (PBS activation is followed by LR initiation and patterning).

Under normal growth conditions, *Arabidopsis* plants display a regularly spaced pattern of LR along the primary root axis. The locations from which LR emerge is controlled by a mechanism called the Root Clock. The Root Clock operates in a region called the Oscillation Zone (OZ) and its activity can be visualized by the synthetic auxin signaling reporter *DR5: Luciferase* (*DR5;* Moreno-Risueno et al., 2010). *DR5* monitoring in the OZ reveals an oscillatory expression pattern every 4–6 h. Those cells that experience a peak of *DR5* expression in the OZ will end up forming a PBS, which shows permanent expression of *DR5* and from which LR emerge. This process is known as priming. Interestingly, the Root Clock oscillatory dynamics are not exclusive of the primary root, but also observed in LR (Figure 1A). Thus, the Root Clock prepatterns the bulk of the root system. Transcriptome analyses of the OZ revealed a complex oscillatory transcriptional response comprising genes that oscillate in phase with *DR5*, and genes that oscillate in anti-phase to *DR5*. Thus, the primary output of the Root Clock was described as the periodic generation of gene expression oscillations to generate PBS (Moreno-Risueno et al., 2010).

New activities and reporters have been recently associated with the Root Clock. The merocyanine aldehyde (MCA) chemical reporter, which measures the retinoid binding protein activity, shows a similar spatial–temporal pattern as *DR5* in the OZ (Dickinson et al., 2021). Intriguingly, simultaneously tracking experiments showed a temporal shift of expression between MCA and *DR5* reporters. MCA activity appeared about 5 h earlier than *DR5;* however, its activity also predicted the locations where PBS were formed (Dickinson et al., 2021).

Within a PBS, the LR organogenesis process is initiated in several xylem pole pericycle (XPP) cells that are specified as founder cells (FCs). Toward LR formation, adjacent FCs will undergo a series of tightly regulated divisions to end up forming a functional organ (Malamy and Benfey, 1997). Most LR organogenesis stages require auxin signaling (Dubrovsky et al., 2008; De Rybel et al., 2010; Lavenus et al., 2013). Recent studies indicate that LR organogenesis might start rapidly after PBS formation as the early LR primordia (LRP) marker *pCLAVATA3/ESR-RELATED44(CLE44)::GFP* is active in most of the newly formed PBS (Wachsman et al., 2020). Therefore, *pCLE44::GFP* emerges as a PBS reporter enabling fluorescent/confocal microscopy of the Root Clock activity, although the function of *CLE44,* which encodes for a small peptide, is unknown in roots.

As the primary root grows, the root exhibits a characteristic wavy pattern. Intriguingly, it exists a high correlation between the root bends and the PBS (Laskowski et al., 2008; Moreno-Risueno et al., 2010). Analyses of *DR5* showed that the oscillations appeared before root bending, suggesting that changes in the root growth direction and the wavy pattern could be an output of the Root Clock (Moreno-Risueno et al., 2010). Although it has been proposed that the root growth dynamics could drive LR priming in a non-dependent clock mechanism (Van Den Berg et al., 2021), it was previously shown that roots with reduced or variable growth have similar PBS number (Moreno-Risueno et al., 2010). Furthermore, the Root Clock impacts the root hormonal state (Perianez-Rodriguez et al., 2021) that could indirectly modify root growth. Future experiments will address if an increase in the Root Clock activity could accelerate root growth.

It has been recently reported that vesicle trafficking mediated by GNOM is required for the Root Clock function and PBS formation. Notably, no observable PBS were found in the *gnom-184* mutant (Wachsman et al., 2020). It is unknown if impaired Root Clock oscillations in the OZ of *gnom-184* caused the absence of PBS, or the PBS could be temporarily formed and lost. The vesicle trafficking mediated by GNOM is involved in cell wall loosening and regulates the levels and distribution of esterified- and de-esterified pectin, which is necessary for LR formation and emergence (Wachsman et al., 2020). As GNOM is required for both PBS formation and esterified−/de-esterified pectin distribution, it is tempting to speculate if the Root Clock would activate the GNOM pathway to mark PBS through the esterification/de-esterification of pectin.

## Inner Machinery of the Root Clock

To ensure the cyclic pattern of PBS throughout the root, the inner machinery of the Root Clock must precisely control the periodicity and amplitude of the oscillations in the OZ. The Root Clock periodicity can be defined as the time between two consecutive pulses of cyclic gene expression (in-phase/anti-phase genes) in the OZ. The amplitude of the Root Clock can be defined as the maximum variation between the higher and lower levels of gene expression during a period of the oscillations.

The mechanism controlling the periodicity was unknown until recently (Figure 1B; Perianez-Rodriguez et al., 2021). The hormone auxin plays a central role in the inner machinery of the Root Clock (De Rybel et al., 2010; Perianez-Rodriguez et al., 2021). Aux/IAA proteins are inhibitors of auxin response factors (ARFs). The presence of auxin releases ARF activity by targeting the Aux/IAAs proteins for degradation by the proteasome (Luo et al., 2018). ARF7 has been proposed to be an activator of auxin response genes (Ulmasov et al., 1999). Thus, releasing the ARF7 inhibition by some Aux/IAA during LR initiation and formation leads to the transcription of auxin response genes (Ulmasov et al., 1999; Weijers et al., 2005; Lavenus et al., 2013). In addition to the role of ARF7 as an activator of auxin response during LR formation, ARF7 has been shown to be a negative regulator of the oscillation periodicity (Perianez-Rodriguez et al., 2021). *DR5* expression in the OZ of the loss-of-function mutant *arf7-1* shows higher levels than control plants and lacks its characteristic oscillatory dynamics. As a result, non-even time-spaced PBS are observed in *arf7-1* (Perianez-Rodriguez et al., 2021). Moreover, a gain of function mutation was identified in the Aux/IAA protein IAA18/POTENT (*iaa18*/*potent* mutant) that mimicked the lack of *DR5* oscillatory dynamics and altered PBS patterning observed in *arf7-1*. Both ARF7 and IAA18/POTENT interact *in vivo* in the OZ to form auxin-dependent heterodimers restricting ARF7 activity (Perianez-Rodriguez et al., 2021). It has been shown that auxin-dependent post-translational modification (SUMOylation) of ARF7 is required for its interaction with IAA3 (Orosa-Puente et al., 2018). Although ARF7-IAA18/POTENT dimer formation does not require SUMOylation (Perianez-Rodriguez et al., 2021), it is possible that other post translational modifications modify ARF7-IAA18/POTENT dimer formation.

Transcriptome analyses of the OZ of *iaa18/potent* and *arf7-1* mutants showed a large percentage of deregulated oscillating genes shared by both mutants. Most of the in-phase deregulated genes were activated in *iaa18/potent* and *arf7-1* whereas most of the antiphase-deregulated genes were repressed. These results indicate that ARF7 is a negative regulator of the in-phase genes and an activator of the antiphase genes, being this function modulated by IAA18/POTENT. Intriguingly, gene ontology analysis of the deregulated genes indicated a role for IAA18/POTENT and ARF7 in the regulation of auxin biosynthesis in the OZ. Further analysis using the biosensor R2D2, which serves as a proxy for auxin concentration, revealed that *arf7-1* and *iaa18/potent* mutants had higher levels of auxin in the OZ, while the ARF7 overexpression presented lower (Perianez-Rodriguez et al., 2021). Thus, the IAA18/POTENT-ARF7 module regulates the periodicity of the oscillations by, respectively, activating and repressing the in-phase and antiphase genes, while it feeds back in auxin levels in the OZ.

The generation of PBS also depends on the amplitude of the oscillations. The auxin response in the gain-of-function mutant *iaa28-1* is reduced, which decreased the oscillations and caused defects in founder cell specification and LRP formation (De Rybel et al., 2010). A possible interpretation is that the IAA28 signaling cascade controls the amplitude of the oscillations in the OZ ensuring the minimum auxin/in-phase gene response threshold to form a PBS. Interestingly, the increased in-phase/*DR5* response in the OZ of *potent* and *arf7-1* mutants decreases when *iaa28-1* is introgressed (Perianez-Rodriguez et al., 2021). This result indicates that IAA28 would act upstream of IAA18/POTENT-ARF7 to generate an oscillation. The increased *DR5* and in-phase gene expression observed in the OZ of *potent* and *arf7-1* can then be attributed to signaling through the IAA28 module to establish the oscillation amplitude. Consistent with the importance of reaching an oscillation amplitude threshold to form a PBS, mutants in genes regulating indole-3-butyric acid (IBA)-to-auxin conversion show reduced oscillation amplitude and PBS formation without presenting alterations in the oscillation frequency (Xuan et al., 2015). All these results highlight the importance of establishing the correct oscillation amplitude. Thus, the core molecular mechanism driving the Root Clock periodicity relies on auxin-dependent negative regulatory loops (Figure 1B), which are simultaneously signaled through IAA28 to establish the in-phase gene expression amplitude and through IAA18/POTENT and ARF7 to generate the periodic oscillatory behavior and fine-tune the oscillation amplitude (Perianez-Rodriguez et al., 2021).

Simulations of the Root Clock oscillatory circuit predict the existence of an unknown factor (F) promoting ARF7-IAA18/POTENT dimer formation in response to auxin (Figure 1B). *ARF7* is an antiphase gene, although the nature of its oscillatory behavior is unknown. As auxin cannot significantly regulate *ARF7* expression in the OZ (Perianez-Rodriguez et al., 2021), if F were an in-phase gene, the Root Clock circuit is predicted to drive autonomous oscillations independently of the *ARF7* oscillations. Furthermore, the *ARF7* antiphase oscillations can also be explained through regulation by an in-phase gene. Consistent with these predictions, mutants in some of in-phase genes such as in the MADS-box transcription factor *SHATTERPROOF (SHP)* genes (*shp1shp2* double mutant) showed altered PBS formation and root bending (Moreno-Risueno et al., 2010), although *SHP1/2* genes have not been shown to be activated by auxin.

## New Insights Into the Root Clock Machinery Positioning

LR prepatterning and the Root Clock show similarities with somitogenesis and the Segmentation Clock of vertebrates, as both mechanisms show gene expression oscillations propagating through a growing axis (Moreno-Risueno and Benfey, 2011; Linde-Medina and Smit, 2021). Interestingly, the determination of somites could be caused by the interaction of the Segmentation Clock with a specific threshold of a gradient of factors called the determination front (Aulehla et al., 2008).

ARF7 is a component of the Root Clock (Perianez-Rodriguez et al., 2021). *arf7-1* mutant shows more PBS and more FCs (Moreno-Risueno et al., 2010; Perianez-Rodriguez et al., 2021); however, it is impaired in subsequent LR initiation and formation steps (Lavenus et al., 2013), indicating (Perianez-Rodriguez et al., 2021) that ARF7 is a repressor of the in-phase oscillations and an activator of LR organogenesis. It is tempting to speculate that the bimodal (repressor/activator) role of ARF7 in roots could be associated with a gradient with similarities to the Determination Front of somitogenesis. We treated roots with auxin and analyzed *DR5* expression in *arf7-1* and the wild type (Figure 1C). *arf7-1* mutant showed higher *DR5* expression in the OZ prior treatment, as expected. Strikingly, *DR5* was activated only in control roots but not in *arf7-1* in the root shootward region above the OZ, suggesting that acquisition of PBS competence to initiate LR organogenesis is determined through an auxin gradient involving ARF7 activity. Future experiments will address if the interaction between the auxin gradient and ARF7 in roots shows similarities with the Determination Front of somitogenesis.

## The First Tier of Root Clock Regulation: Endogenous Cues

The Root Clock shows a stable oscillatory pattern that can compensates for certain changes in temperature (18–24°C range), photoperiod/continuous light and sucrose content (Moreno-Risueno et al., 2010). In contrast, the root system shows a plastic developmental capacity, which has been associated with different physiological states and management of the plant energetic resources (Xuan et al., 2020).

In agreement with the possibility of the Root Cock being able to respond to different physiological states or endogenous signals, it has been shown that the seedling age influences PBS formation. Changes in the number of PBS per root are a proxy for changes in the Root Clock oscillatory frequency. The number of PBS formed for the first 2 days after germination was low, increasing during next 4 days and stabilizing for the rest of the days analyzed (Xuan et al., 2015). As the plant develops, the metabolism increases resulting in higher hormonal levels. A possible hypothesis is that changes in the *DR5* oscillatory frequency are due to variations in the hormonal state of the plant during development.

The hormonal state of the plant influences the root system architecture (Chaiwanon et al., 2016). Auxin stands out as a key hormone for root development; in addition, it is involved in the generation of the oscillations (Lavenus et al., 2013; Xuan et al., 2016; Laskowski and Ten Tusscher, 2017; Perianez-Rodriguez et al., 2021). As the root grows, programmed cell death (PCD) of the lateral root cap (LRC) cells has been described to cyclically occur at the onset of the elongation zone. Repeated bursts of cell death have been proposed to stimulate conversion of IBA to auxin. As a result pulses of auxin could be released into the primary root coinciding with the establishment of PBS (Kumpf and Nowack, 2015; Xuan et al., 2015, 2016). Interestingly, both more and less PCD inhibited the oscillatory pattern (Xuan et al., 2016). Even though auxin is necessary to establish a PBS, exogenous auxin application in the OZ only generated a PBS when it was coincident with an in-phase gene oscillation (Moreno-Risueno et al., 2010), which can be explained by the structure of the Root Clock oscillatory circuit (Perianez-Rodriguez et al., 2021). Thus, it can be speculated that auxin pulses generated by PCD are required for entrainment of the Root Clock.

Auxin levels influence root growth and require auxin transport, which is mediated by the arrangement of specific influx and efflux carriers (Motte et al., 2019; Xuan et al., 2020). Notably, *in silico* simulations showed that the effect of auxin influx on priming is specific to the OZ, while repetitive pulses of auxin can entrain the Root Clock and change the oscillatory frequency (Perianez-Rodriguez et al., 2021). Thus, variation of auxin levels would modify both PBS formation and the primary root growth, thereby facilitating their coordination and optimizing root system architecture. Morphological factors can influence auxin accumulation (Van Den Berg et al., 2021). An example is gravistimulation, which causes transient accumulation of auxin in the OZ. Notably, the alteration of the primary root growth following changes of 180° in the gravity vector reduces the time interval between the oscillations that increase PBS formation. (Moreno-Risueno et al., 2010; Band et al., 2012; Xuan et al., 2016; Perianez-Rodriguez et al., 2021).

IBA-derived auxin contributes to priming and LR formation (Xuan et al., 2015). However, whether IBA has a role independently of auxin remains under discussion. Recently, TRANSPORTER OF IBA 1 (TOB1) has been described to regulate IBA accumulation in the vacuoles of specific cells. As cytokinin transcriptionally regulates TOB1 (Michniewicz et al., 2019), this transporter could integrate cytokinin and auxin responses to regulate the Root Clock oscillations.

The carotenoids, a family of metabolites different from auxin-related compounds, have also been discovered to affect the Root Clock. A chance observation showed that Arabidopsis seedlings treated with carotenoids biosynthesis inhibitors were impaired in LR formation. A more detailed analysis revealed that LR prepatterning was inhibited in plants treated with norflurazon and 2-(4-chlorophenylthio)-triethylamine hydrochloride (CPTA). These compounds inhibit key enzymes in the carotenoid biosynthetic pathway, indicating a role for carotenoids or their derivatives in the regulation of the Root Clock (Van Norman et al., 2014). Several apocarotenoids, such as abscisic acid, strigolactones, and β-cyclocitral, regulate root development (De Smet et al., 2003; Van Norman et al., 2014; Jiang et al., 2016; Dickinson et al., 2019). However, preventing the Root Clock oscillations through the carotenoid cleavage inhibitor D15 was not rescued by treatment with these apocarotenoids (Van Norman et al., 2014; Dickinson et al., 2019). Additionally, strigolactones have a slight negative effect on LR priming (Jiang et al., 2016). These results pointed out that the Root Clock oscillations should be regulated by a yet uncharacterized carotenoid derivative.

A more recent metabolomic approach showed that the concentration of several retinoids specifically decreased when the carotenoid metabolism was blocked using D15. Furthermore, treatment with these retinoids, particularly retinal and its precursor apo14, could rescue the *DR5* oscillations and LR formation (Dickinson et al., 2021). This result indicates a role for retinal signaling in the regulation of the Root Clock. Temperature-induced lipocalin (TIL), previously described in thermotolerance response (Chi et al., 2009), was identified as a retinal interactor. Interestingly, TIL does not appear to interact with other structurally similar apocarotenoids, suggesting high specificity. Additionally, *til* mutants show a reduction in the *DR5* oscillation amplitude and less LR branching, suggesting that TIL regulates retinal signaling to establish the Root Clock oscillations and PBS formation (Dickinson et al., 2021). Based on these data, it can be hypothesized that this factor is responsible for the communication between carotenoid metabolism and the inner machinery of the Root Clock, thereby facilitating the integration of retinal signaling by the oscillatory circuit. To date, it still needs to be determined how TIL interacts with the Root Clock.

Taken together, all this information illustrates how the physiological state of the plant, i.e., the hormonal state and secondary metabolites, can modify or entrain the Root Clock oscillator. As in other biological clocks, this entrainment makes the peak of one of the oscillation types (the in-phase oscillations) coincide with an internal cue. An additional effect is that signals with lower frequency than the Root Clock oscillations change the period, as observed during gravistimulation (Moreno-Risueno et al., 2010). If some of these internal cues are also outputs of the Root Clock, forming feedback loops (similar to auxin) is still an open question.

## The Second Tier of Root Clock Regulation: Exogenous Cues

The root system adapts to changing environmental conditions, such as nutrient and water availability, abiotic stress, etc. (Motte et al., 2019). Signals coming from the external environment also affect the Root Clock activity, although direct interactions have not been reported yet (Kircher and Schopfer, 2018; Orman-Ligeza et al., 2018; Xie et al., 2019; Dickinson et al., 2021; Duan et al., 2021).

Light is one of the external factors influencing the Root Clock activity. It has been shown that in absence of light, roots show reduced and irregular formation of PBS, which are not persistent and fail to generate LRP. This effect can be reversed by light or auxin (1-naphthalene acetic acid—NAA) supplementation (Kircher and Schopfer, 2018). Using pharmacological inhibitors, this study showed that the tryptophan-dependent auxin biosynthesis pathway is responsible of the effect of light on the Root Clock. A more recent study showed that the heme oxygenase LONG HYPOCOTYL1 (HY1), involved in phytochrome morphogenesis (Koornneef and Kendrick, 1994), mediates the effect of light on the Root Clock. Light-regulated *HY1* expression and *hy1* mutants showed a reduction in the *DR5* oscillation amplitude and in PBS formation. Interestingly, grafting experiments showed that this effect is primarily due to HY1 function in the shoot, although root-derived HY1 also has some influence (Duan et al., 2021). HY1 is not a mobile factor and it was shown that HY1 regulates the expression of *HY5* and its homolog *HYH* to maintain expression and intracellular localization of the auxin transporters AUX1, PIN1, and PIN2 in roots. In turn, these transporters are required for auxin accumulation in the OZ (Duan et al., 2021). Thus, the effect of light on the Root Clock activity occurs indirectly through modification of auxin biosynthesis and/or its distribution.

**Figure 2:**
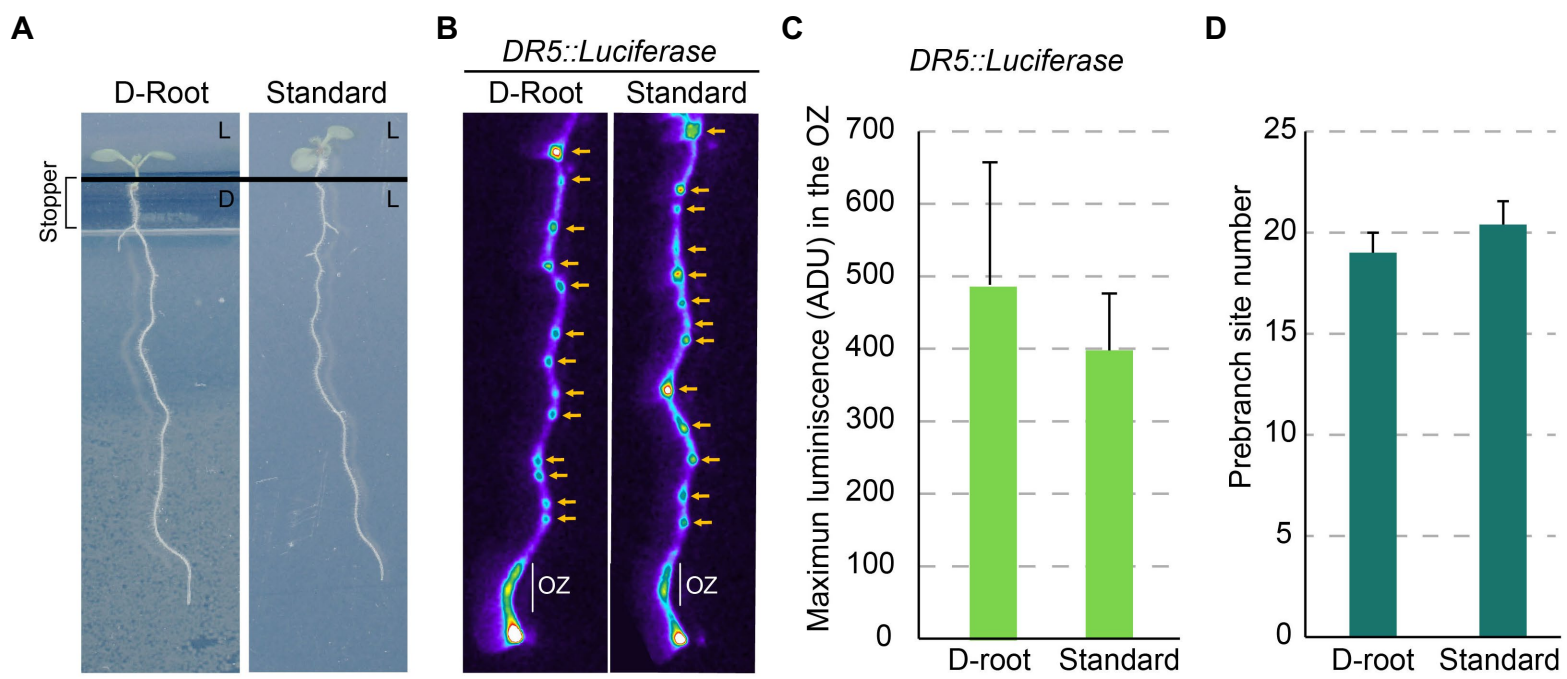
Light perceived in the root does not affect the Root Clock activity. **(A)** 7 dpi plants grown in the D-Root system and under standard conditions. L: plate section exposed to light, D: plate section maintained in the dark. **(B)** *DR5::Luciferase* imaging of roots grown in the D-Root and under standard conditions. Yellow arrow: PBS. **(C)** *DR5::Luciferase* quantification in the OZ of roots in the D-Root and under standard conditions. ADU: Analog-Digital Units **(D)** Number of PBS formed at 7 dpi in the D-Root and under standard conditions. No statistical significance by Student’s *t*-test (value of *p* <0.05) was observed (*n* = 10).

A remaining question is whether the perception of the light by roots affects the Root Clock. We performed an experiment to visualize *DR5* using the D-root system (Silva-Navas et al., 2015). In the D-root the roots grow in darkness, while the shoots are exposed to light (Figure 2A). We did not find obvious differences in the expression of *DR5* in the OZ or in the number of PBS of seedlings grown in the D-root system compared with seedlings grown with both the shoot and root under light (Figures 2B–D). These results indicate that light in the root does not change the amplitude or frequency of the Root Clock oscillations.

Local water availability could also impact the Root Clock. It has been reported that the local contact of root with water can activate formation of LRs in those regions. In contrast, FC located in root regions not in contact with water are arrested in development. The resulting mechanism called hydropatterning regulates sidedness of the LR (Bao et al., 2014). Notably, hydropatterning affects the distribution of the in-phase gene *LATERAL ORGAN BOUNDARIES DOMAIN 16 (LBD16)* in an ARF7-dependent manner (Orosa-Puente et al., 2018), while *LBD16* was first described to regulate LR initiation (Goh et al., 2012). Additional experiments will determine the effect of hydropatterning on the Root Clock. The Root Clock might indirectly affect LR sidedness as it regulates root bending and LR locate on the outside of the root bends (Laskowski et al., 2008; Moreno-Risueno et al., 2010). Pectin esterification/de-esterification might also affect LR sidedness (Wachsman et al., 2020), and it can be hypothesized to be an output of the Root Clock as previously commented. Thus, it is tempting to speculate if the Root Clock would impact LR sidedness through pectin esterification/de-esterification while being, in turn, affected by hydropatterning.

In a different study, it was shown that LR formation is repressed when roots penetrate soil air spaces and lose contact with water. This process is called xerobranching and is triggered by the accumulation of abscisic acid (ABA) in the root. ABA treatments mimicked the xerobranching response and they generated a reduction in the *DR5* oscillations and PBS formation (Orman-Ligeza et al., 2018). ABA accumulation could decrease free auxin levels in the root, as supplementation with auxin recovered the oscillations and PBS formation in ABA pretreated plants. However, it is also possible that ABA accumulation during xerobranching occurs because of an alteration in auxin signaling.

Several studies have shown that cadmium inhibits LR formation, which can be attributed to the alteration of auxin homeostasis and signaling (Hu et al., 2013; Yuan and Huang, 2016). Recently, it has been reported that cadmium interferes with the *DR5* oscillations (reducing their frequency) and with PBS formation (Xie et al., 2019). Cadmium treatments repressed cell cycle activity in the meristem, which might affect the load of auxin required for priming (Van Den Berg et al., 2021). Most importantly, cadmium inhibits auxin homeostasis and signaling (Hu et al., 2013; Yuan and Huang, 2016) which can impact in the Root Clock oscillatory circuit leading to the observed reduction in the *DR5* oscillatory frequency. Cadmium also reduced the number of LRC cells and the frequency of LRC PCD pulses, which can impact the *DR5* oscillatory frequency.

The effect of cadmium treatments was also tested in *SOMBRERO (SMB)* mutants. *SMB* is a regulator of root cap cellular maturation (Bennett et al., 2010) and PCD required for Root Clock activity and LR priming (Xuan et al., 2016). Interestingly, this mutant was hyposensitive to cadmium stress, suggesting a link between the effects of cadmium and the alteration of the frequency of the LRC PCD pulses and LR priming (Xie et al., 2019).

Temperature has been recently shown to alter the Root Clock by reducing the amplitude of the *DR5* oscillations when plants were exposed to heat shock (Dickinson et al., 2021). This process occurs through TIL, a lipocalin previously described to bind retinal and to be required for activity of the Root Clock. Therefore, it is possible that TIL integrates exogenous cues such as heat into the Root Clock machinery. Whether the heat shock response occurs through the alteration of retinal signaling is a question that still needs to be addressed.

These results show that regulation of the Root Clock by the environment occur indirectly through the modification of the endogenous cues directly interconnected with the Root Clock machinery. Hence, the responses of the Root Clock to light, water deprivation, and cadmium would be mediated by modification of auxin homeostasis and signaling, while regulation of the response to heat might involve retinal signaling.

## The Root Clock as a Signal Integrator System and Future Perspectives

**Figure 3:**
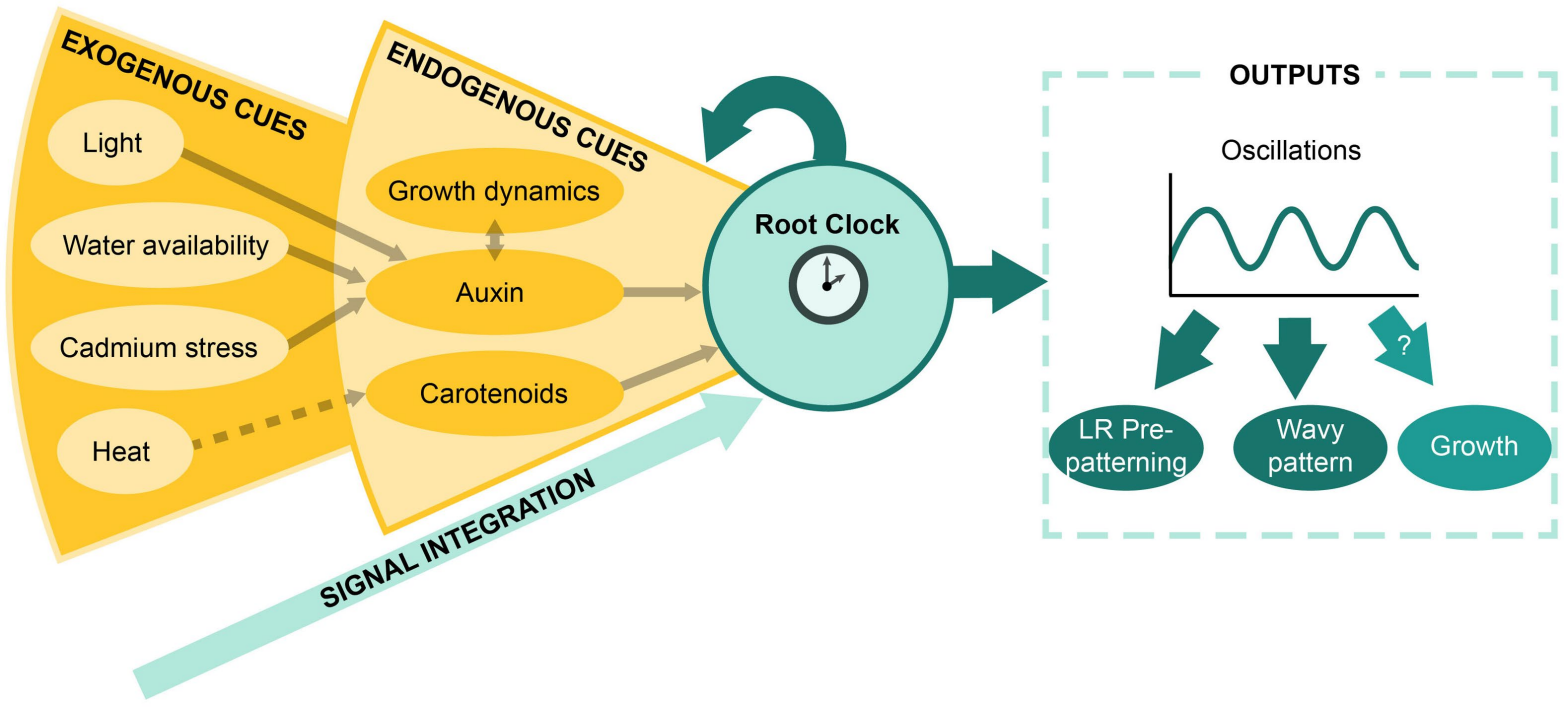
The Root Clock as a signal integrator system. Schematic representation of the model proposed for the integration of exogenous and endogenous signals by the Root Clock and the resulting outputs.

Altogether, we have highlighted that the outputs of the Root Clock, such as the oscillations in gene expression and PBS establishment, result from the activity of an inner oscillatory circuit (Moreno-Risueno et al., 2010; Perianez-Rodriguez et al., 2021). Additionally, the Root Clock can directly integrate endogenous signals such as the hormonal state (primarily auxin) and carotenoid metabolism (Figure 3; Xuan et al., 2015, 2016; Jiang et al., 2016; Dickinson et al., 2021; Perianez-Rodriguez et al., 2021), which function as entrainment signals and/or modifiers of the oscillatory dynamics. In turn, the Root Clock could feed back into these endogenous cues. The Root Clock can also indirectly respond to environmental cues. Proven examples include the adaptation to light and water availability, as well as the exposition to cadmium and heat (Orman-Ligeza et al., 2018; Xie et al., 2019; Dickinson et al., 2021; Duan et al., 2021). Although, most of the environmental changes interfere with the Root Clock activity through the modification of auxin, heat shock might be related to an alteration in retinal signaling. In support of this idea, exposition to high temperatures is shown to reduce carotenoid biosynthesis in tomato plants (Bianchetti et al., 2020).

Plant species with a tap root system present regular-spaced pattern of LRs as observed in *Arabidopsis* (Chen et al., 2018). Whether these species present oscillatory gene expression in the OZ remains unknown. In support of conservation of the Root Clock mechanism in dicotyledonous plants, the *DR5* synthetic promoter shows a similar expression pattern in poplar (Chen et al., 2013) compared with Arabidopsis. Monocotyledonous plants present a fibrous system primary formed by many adventitious roots. As the Root Clock is not only present in the PR but also in LR (Figure 1A), a Root Clock mechanism cannot be discarded in the adventitious roots of monocots. It would be interesting to introgress the Root Clock reporters (Moreno-Risueno et al., 2010; Wachsman et al., 2020; Dickinson et al., 2021; Perianez-Rodriguez et al., 2021) in dicot and monocot species to investigate whether the Root Clock is conserved across the plant kingdom.

LR prepatterning and the Root Clock show similarities with somitogenesis and the Segmentation Clock of vertebrates. Future experiments will reveal if a determination front is required for initiation of LR organogenesis from PBS. Furthermore, if a determination front analogous to somitogenesis existed, PBS formation would be the result of the interaction of the in-phase oscillations with a gradient establishing a limit between the OZ and the region of PBS formation. In agreement with this possibility, we observed a boundary between the OZ and the root shootward region where PBS are activated to initiate LR organogenesis (Figure 1C).

## Experimental Procedures

### Plant Material and Growth Conditions

*Arabidopsis thaliana* seeds were sterilized with gas chlorine (1% HCl) in a confined environment for 2 h. After stratification for 3 days at 4°C, plants were grown on 12 × 12 cm plates with Murashige and Skoog basal medium (2,2 g/l MS, 0.05% MES, 1% sucrose, 1% plant-agar) in a chamber with 16/8-h light/dark photoperiod at 21–23°C. To address the contribution of root illumination to the Root Clock dynamics, we took advantage of the D-Root system, previously described by Silva-Navas et al. (2015). *Columbia (Col-0)* and *arf7-1* mutant both carrying the *DR5::Luciferase* reported are those in (Perianez-Rodriguez et al., 2021). Plants were analyzed at 6 or 7 days post-imbibition (dpi) as indicated in the figure legends.

### Chemical Treatments

Seedlings were grown on MS/2 plates and transferred at 6dpi to plates containing 0.25 μm indole-acetic acid (IAA) for 1.5 h before luciferase imaging.

### Luciferase Imaging and Quantifications

Plates were sprayed with 1 ml of 2.5 mm potassium luciferin (Gold Biotechnology, St. Louis, Mo., cat.no: LUCK-1), incubated for 3 min, and then imaged using a NightOwl II (Berthold) with an exposure time of 5 min. Images were analyzed using MetaMorph Image Analysis Software. To measure *DR5::Luciferase* expression levels, region of interest (ROI) was selected over the OZ and the analog-digital units (ADU) per pixel quantified. The number of PBS was determined in MetaMorph through quantification of the number of sites with high relative expression through the primary root.

### Statistical Analysis

An independent samples Student’s *t*-test was used for statistical evaluations using value of *p* < 0.05. Number of replicates is 10 in all experiments.

## Author Contributions

MM-R, EB-A, and LS-R contributed to conceptualization and writing—review. EB-A and LS-R contributed to methodology and writing—original draft. MM-R contributed to resources, editing, supervision, and funding acquisition. All authors contributed to the article and approved the submitted version.

## Funding

This work was funded by Ministerio de Economía y Competitividad (MINECO) of Spain (grant PID2019-111523GB-I00 to MM-R) and by the “Severo Ochoa Program for Centres of Excellence in R&D” from the Agencia Estatal de Investigacion of Spain [SEV-s-0672 (2017-2021) and CEX2020-000999-S-21-1] to MM-R through CBGP. LS-R was supported by FPI contract BES-2014-068852 from MINECO and EB-A by contract associated to Programa Atraccion de Talento from CM (2017-T2/BIO-3453, PI: P. Perez-Garcia).

## Conflict of Interest

The authors declare that the research was conducted in the absence of any commercial or financial relationships that could be construed as a potential conflict of interest.

## Publisher’s Note

All claims expressed in this article are solely those of the authors and do not necessarily represent those of their affiliated organizations, or those of the publisher, the editors and the reviewers. Any product that may be evaluated in this article, or claim that may be made by its manufacturer, is not guaranteed or endorsed by the publisher.
